# Successful Surgical Excision of a Chronic Abscess in a Hawksbill Turtle (*Eretmochelys imbricata*): A Case Report

**DOI:** 10.3390/vetsci12121172

**Published:** 2025-12-09

**Authors:** Da Sol Park, Hee Jun Ko, Jiho Park, Sib Sankar Giri, Mae Hyun Hwang, Jae Hong Park, Eun Jae Park, Se Chang Park

**Affiliations:** 1 Laboratory of Aquatic Biomedicine, Research Institute for Veterinary Science, College of Veterinary Medicine, Seoul National University, Seoul 08826, Republic of Korea; dabol2@snu.ac.kr (D.S.P.); hjk9538@gmail.com (H.J.K.); ssgiri@snu.ac.kr (S.S.G.); ghkdao@snu.ac.kr (M.H.H.); jaehong139@snu.ac.kr (J.H.P.); eunjae.p@snu.ac.kr (E.J.P.); 2 Lotte World Aquarium, Seoul 05551, Republic of Korea; 3 Haneul Veterinary Clinic, Incheon 22371, Republic of Korea; skycity0075@naver.com; 4 Faculty Startup Company SHA Bio Co., Ltd., Research Institute for Veterinary Science, College of Veterinary Medicine, Seoul National University, Seoul 08826, Republic of Korea

**Keywords:** reptile surgery, marine reptile, aquatic veterinary medicine, case report

## Abstract

In sea turtles, soft tissue abscesses typically present with a solid, caseous consistency and respond poorly to pharmacological treatment. A hawksbill turtle (*Eretmochelys imbricata*) exhibited a chronic subcutaneous mass near the cloacal region that had persisted for three years. Histopathological examination confirmed the existence of an abscess composed of concentric necrotic layers and chronic inflammatory cell infiltration, with no detectable pathogens. The lesion was surgically excised under local anesthesia with a radiofrequency electrosurgical unit, minimizing bleeding and operation time. Postoperative management consisted of oral antibiotic administration to minimize handling stress. The turtle recovered uneventfully, with no complications or recurrence during the two-month follow-up period. This case highlights that chronic abscesses in sea turtles require surgical excision as the most effective therapeutic approach and provides practical insights for marine reptile medicine.

## Simple Summary

Sea turtles often develop soft tissue abscesses, which are swellings caused by infection or inflammation and are usually very firm with little or no pus inside. Because of this structure, they do not respond well to pharmacological treatment and often persist for long periods. In this report, a hawksbill turtle (*Eretmochelys imbricata*) had a chronic lump near the cloaca that lasted for three years. Microscopic examination showed layers of dead tissue and immune cells, but no obvious pathogens. The mass was removed with a surgical procedure performed under local anesthesia, which reduced stress and bleeding. After surgery, the turtle was treated with oral antibiotics and recovered without complications. This case shows that chronic abscesses in sea turtles are best managed by surgical removal rather than medication alone. Sharing this knowledge can help veterinarians improve clinical care for sea turtles and support the conservation of this endangered species.

## 1. Introduction

Rehabilitation of sick and injured marine animals has become a fundamental component of conservation efforts for threatened or endangered species and biodiversity preservation worldwide [1,2]. As the clinical management of marine animals gains increasing attention, a broader understanding of their common diseases is essential to improve outcomes in both rehabilitation and captive care [3].

Abscesses are frequently observed in sea turtles and may develop due to various causes, including ectoparasite attachment, puncture wounds, foreign bodies, injection sites, poor environmental hygiene, and dermatophilosis [4]. These abscesses are often encountered in reptiles as firm, caseous lesions rather than the fluid-filled abscesses typically observed in mammals. Unlike mammals, reptiles lack the lysozymes necessary to liquefy purulent material, resulting in the formation of hard, inspissated pus that cannot be drained and typically requires surgical excision for resolution [5].

Postoperative infection control is particularly challenging in marine animals. Surgical sites in aquatic species remain continuously exposed to seawater, which contains various opportunistic pathogens. Nevertheless, animals are often returned to the water shortly after surgery to minimize stress and encourage feeding, even when wound healing is incomplete [6].

Careful consideration of surgical techniques that minimize intraoperative bleeding and shorten surgical time may contribute to reducing the risk of infection in these cases. This case report describes the simple excision of a subcutaneous mass near the cloacal region in a hawksbill turtle (*Eretmochelys imbricata*). This case may serve as a practical clinical reference for the management of similar soft tissue lesions in marine animals.

## 2. Case Description

**Figure 1:**
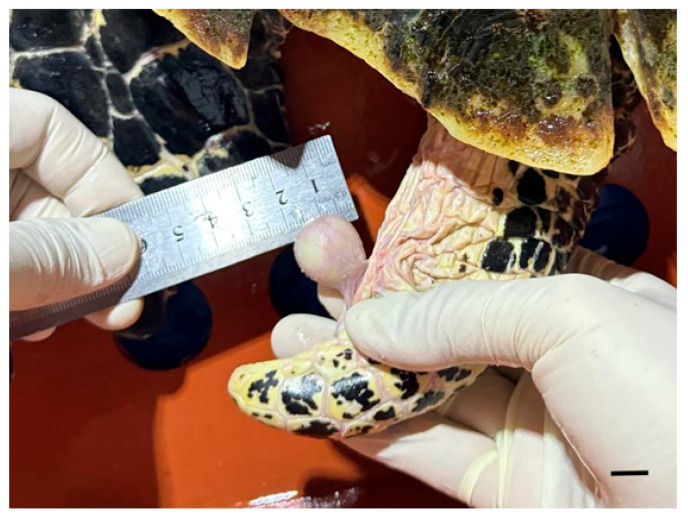
Subcutaneous mass located near the cloacal region of a hawksbill turtle (*Eretmochelys imbricata*). The lesion appeared as a clearly demarcated, round subcutaneous mass in the ventral cloacal area (bar = 1 cm).

An adult male *Eretmochelys imbricata* housed at Lotte Aquarium (Seoul, Republic of Korea) in a closed recirculating marine system (salinity 31‰, temperature 25 °C) was presented with a subcutaneous mass near the cloacal region (Figure 1). The turtle had a curved carapace length of 80 cm, a carapace width of 62 cm, and a body weight of 41.7 kg. The mass was first documented in 2022 and had been present for three years prior to examination.

Throughout this period, the turtle remained bright, alert, and responsive, maintaining normal appetite and activity with no evidence of systemic illness. Surgical intervention was initially deferred because the mass remained small and stable, and the animal’s overall condition was clinically stable. On physical examination, the mass was firm, well-demarcated, and non-fluctuant, with no overlying erythema or discharge. No other abnormalities were detected during a general examination.

Preoperative blood analysis revealed several mild-to-moderate abnormalities, including elevated levels of alkaline phosphatase and total cholesterol, along with decreased alanine aminotransferase (Table 1). Biochemical parameters were measured using an automated dry-chemistry analyzer (FUJI DRI-CHEM NX600; FUJI, Tokyo, Japan). Biochemical variations of this nature have been reported in sea turtles without overt disease and may be associated with dietary factors, physiological status, or minor subclinical changes [7].

White blood cell analysis indicated a relative increase in packed cell volume, eosinophils, lymphocytes, and monocytes, with a corresponding decrease in granulocyte proportion (Table 2). Hematological analysis was performed manually. The observed leukocyte pattern is consistent with a chronic inflammatory process and corresponds well with the long-standing nature of the mass. Other parameters, including electrolytes and glucose, were within acceptable ranges (Table 1).

**Figure 2:**
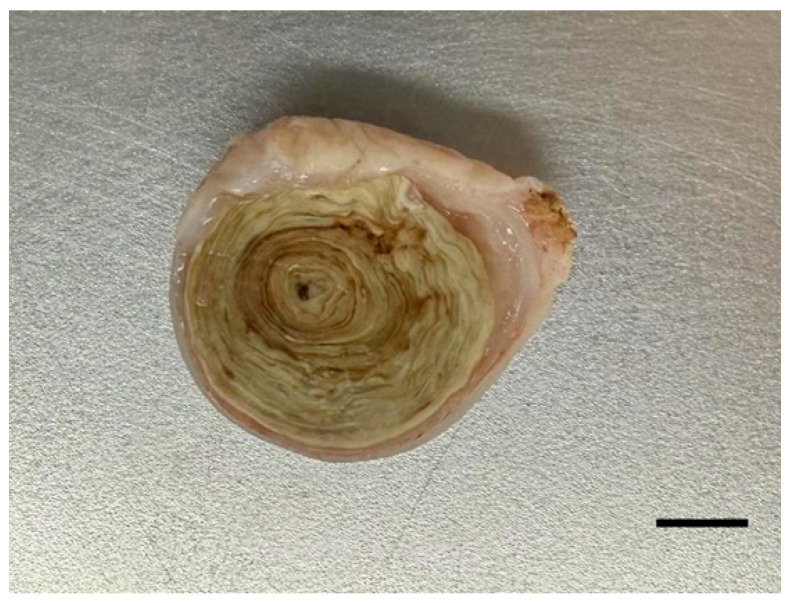
Gross appearance of the excised subcutaneous mass. The cut surface shows a round lesion with a distinct layered structure. The specimen measured 2.0 × 1.8 cm^2^ (bar = 5 mm).

**Figure 3:**
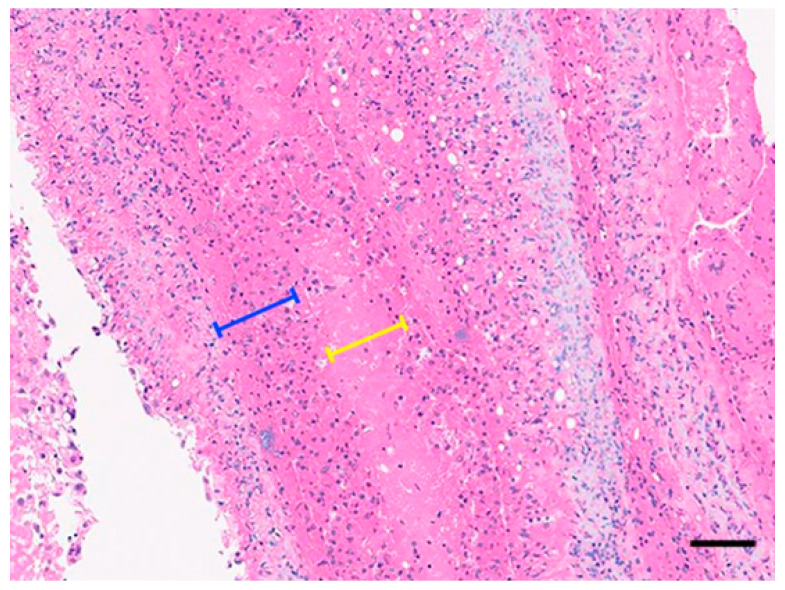
Histological section of the excised mass stained with hematoxylin and eosin (H&E, ×200). The necrotic layer and surrounding inflammatory rim are indicated by bracket lines (yellow and blue, respectively). Bar = 100 µm.

**Figure 4:**
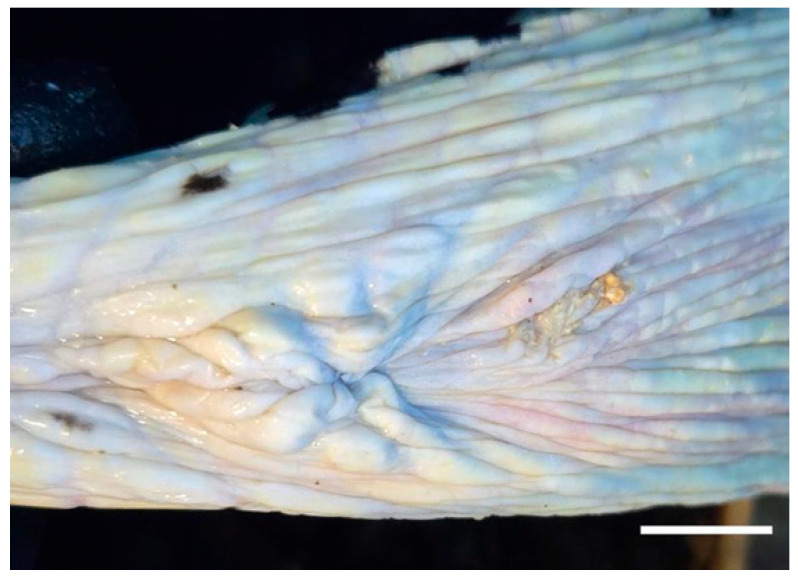
Postoperative image of the surgical site two weeks after the procedure. The surgical area is indicated by an arrow. Bar = 1 cm.

The mass was surgically excised using a radiofrequency electrosurgical unit under local infiltration anesthesia with lidocaine (1 mL, Daehan Lidocaine Hydrochloride Hydrate, Daehan Pharmaceutical Co., Ltd., Seoul, Republic of Korea). Meloxicam (0.2 mg/kg, intramuscular, Metacam, Boehringer Ingelheim Korea Ltd., Seoul, Republic of Korea) was administered postoperatively. Oral medications, including tetracycline (6.1 mg/kg, once daily [SID] for 4 days, Tetracycline Capsule, Chong Kun Dang Pharm Co., Ltd., Seoul, Republic of Korea), meloxicam (0.2 mg/kg, SID for 4 days, CMG Meloxicam Capsule, CMG Pharmaceutical Co., Ltd., Seoul, Republic of Korea), and silymarin (13.7 mg/kg, SID for 4 days, Silymarin Tablet, ELT Science Co., Ltd., Seoul, Republic of Korea), were subsequently prescribed. Ten days postoperatively, mild residual inflammation was suspected, and an additional oral course of lincomycin (6.1 mg/kg, SID for 5 days, Lincomycin Capsule 250 mg, Yuyu Pharma Inc., Seoul, Republic of Korea) and meloxicam (0.2 mg/kg, SID for 5 days, Melocam Capsule, Daehan New Pharm Co., Ltd., Seoul, Republic of Korea) was initiated. The excised mass measured 2.0 cm × 1.8 cm and was firm and well demarcated (Figure 2). Histopathological examination revealed a layered structure composed of fibrin, necrotic debris, and inflammatory cells, without evidence of encapsulation except for dermal fibrous tissue compression (Figure 3). No bacteria or other microorganisms were identified within the lesion. The postoperative course was uneventful, and no recurrence or complications were observed during the two-month follow-up period (Figure 4).

## 3. Discussion

Surgical management in marine animals poses inherent challenges, particularly concerning postoperative infection control. Unlike terrestrial species, aquatic animals are constantly exposed to aquatic environments harboring a variety of opportunistic pathogens, which can significantly increase the risk of surgical site contamination [9]. Furthermore, postoperative protocols in marine species often involve early reintroduction to the aquatic environment to alleviate handling stress and support normal feeding behavior, even though wound healing may not be complete at this stage [6]. These factors complicate postoperative wound management and make achieving an uncomplicated recovery in marine species particularly difficult. Despite advancements in veterinary surgical techniques, significant gaps remain in their application to marine animals, with limited opportunities for veterinarians to accumulate surgical experience in these species. The scarcity of peer-reviewed reports further restricts the dissemination of surgical knowledge in the field of marine mammal and reptile medicine [10]. Given these constraints, individual case reports remain valuable clinical resources for advancing surgical practices in marine animal care.

This case describes the surgical excision of a subcutaneous mass located near the cloacal region in an Eretmochelys imbricata. The mass was firm, well-demarcated, and histopathologically diagnosed as an abscess. The procedure involved surgical excision under local infiltration anesthesia using a radiofrequency electrosurgical unit. This approach minimized intraoperative bleeding and shortened operative time, thereby reducing the risk of contamination in the aquatic environment. Local infiltration anesthesia was selected over general anesthesia to minimize physiological stress and facilitate rapid postoperative recovery. In chelonians, general anesthesia can be challenging due to their unique anatomy and diving physiology, which predispose them to ventilation–perfusion mismatches and respiratory depression [11]. Moreover, the induction and recovery phases of general anesthesia are often prolonged because of temperature-dependent metabolic rates, increasing the risk of delayed recovery and respiratory compromise during emergence from anesthesia [12]. Given the superficial location of the lesion and the brief surgical duration, local anesthesia provided sufficient analgesia while avoiding these risks. Intramuscular antibiotics were administered immediately after the procedure to provide initial infection control. Minimizing stress is particularly important in reptiles as it can compromise immune function and delay wound healing [13]. Therefore, subsequent antibiotic therapy was administered orally rather than by repeated injection to reduce handling stress. Observations by the attending veterinarian at Lotte Aquarium indicated that the turtle resumed normal feeding and swimming behavior shortly after surgery, with no recurrence or complications during the two-month follow-up period.

Histopathological examination revealed a well-demarcated abscess with concentric necrotic layers surrounded by inflammatory cells and fibrous connective tissue, consistent with the chronic abscess pattern commonly observed in reptiles [5]. No microorganisms were identified in the lesion. While the gross and microscopic features were consistent with a chronic abscess, other differential diagnoses, such as cysts, granulomatous inflammation, or neoplasms, were considered. The absence of epithelial lining and cellular atypia, along with the presence of layered necrosis and inflammatory cell infiltration, supported the diagnosis of a chronic abscess rather than these alternative conditions. Although sterile or aseptic abscesses have been described in systemic inflammatory or autoimmune conditions, the present case showed only mild, localized inflammation without systemic abnormalities, making such etiologies unlikely [14]. Abscess formation can arise from multiple factors, such as ectoparasite infestations, penetrating injuries, the presence of foreign bodies, injection-related trauma, suboptimal environmental conditions, and dermatophilosis [15]. Microbiological culture was not performed in this case, but the absence of detectable microorganisms may reflect the presence of a sterile center, as reported in reptilian abscesses, where compression by surrounding fibrous tissue and chronic necrotic changes can eliminate viable bacteria over time [16]. Considering that the lesion had been present for three years, it is plausible that the bacterial load had gradually diminished or had been cleared by the host’s immune response over time. Despite the absence of viable pathogens, the abscess persisted as a chronic, well-formed mass, necessitating surgical excision for resolution. This clinical finding is consistent with previous reports where pharmacological therapy alone was insufficient to resolve subcutaneous abscesses in Kemp’s ridley sea turtle (*Lepidochelys kempii*), requiring surgical intervention for complete removal [17].

While the case resulted in successful resolution, certain limitations should be considered when interpreting the findings. A microbiological culture of the abscess was not performed, which would have allowed confirmation or exclusion of possible anaerobic or fungal pathogens that may not have been detected histologically. Although no microorganisms were identified microscopically, culture or molecular analysis could have provided additional diagnostic certainty. In addition, long-term follow-up has not yet been performed, limiting assessment of possible recurrence or delayed complications. Nevertheless, these limitations do not diminish the clinical relevance of the present case.

This case highlights the successful surgical management of a chronic subcutaneous abscess in an *Eretmochelys imbricata* using a straightforward approach under local anesthesia. Although soft tissue abscesses are relatively common in sea turtles, peer-reviewed reports describing their clinical management and presentation remain scarce. Reporting individual cases like this provides valuable clinical guidance for veterinarians working with marine reptiles. Further accumulation of cases will be essential to refine diagnostic and surgical strategies, thereby broadening their applications in aquatic species.

## Figures and Tables

**Table 1:** Preoperative serum biochemistry of a hawksbill turtle (*Eretmochelys imbricata*). Reference intervals were derived from Tauer et al. [8].

Chemistry Parameters ^1^	Reference	Unit	Measured Value
Sodium	140.7–169.4	mmol/L	156.0
Potassium	3.7–5.7	mmol/L	4.5
Chloride	96.9–148.5	mmol/L	116.0
Calcium	1.0–21.2	mg/dL	8.6
Phosphorus	3.4–16.1	mg/dL	8.7
Uric acid	1.0–1.8	mg/dL	1.5
Total Protein	2.6–5.0	g/dL	3.3
Total Cholesterol	107.1–366.8	mg/dL	1244.0
Glucose	57.4–142.0	mg/dL	134.0
Creatine kinase	121–1296.2	U/L	801.0
AST	18.3–74.3	U/L	50.0
ALT	16.3–78.1	U/L	1.0
ALP	25.7–93.6	U/L	162.0

^1^ AST, aspartate aminotransferase; ALT, alanine aminotransferase; ALP, alkaline phosphatase.

**Table 2:** Hematological profiles of a hawksbill turtle (*Eretmochelys imbricata*). Reference intervals were derived from Tauer et al. [8].

Hematological Parameters ^1^	Reference	Unit	Measured Value
PCV	23–33.6	%	37.0
WBC	1.2–10.8	×10^9^/L	3.9
Heterophils	0–8.5	×10^9^/L	1.2
	43.4–93.6	%	32.0
Lymphocytes	0.08–2.5	×10^9^/L	1.8
	41.8–42.6	%	47.0
Monocytes	0–0.7	×10^9^/L	0.4
	0.3–11.8	%	12.5
Eosinophils	0–0.29	×10^9^/L	0.3
	0–5	%	8.5

^1^ PCV, packed cell volume; WBC, white blood cells.

## Data Availability

The original contributions presented in this study are included in the article. Further inquiries can be directed to the corresponding author.

## Abbreviations

The following abbreviations are used in this manuscript:

AST	Aspartate aminotransferase
ALT	Alanine aminotransferase
ALP	Alkaline phosphatase
PCV	Packed cell volume
WBCs	White blood cells
SID	Once daily

## References

[1] Escobedo-Bonilla C.M., Quiros-Rojas N.M., Rudín-Salazar E. (2022). Rehabilitation of marine turtles and welfare improvement by application of environmental enrichment strategies. Animals.

[2] Simeone C.A., Rousselet E., Atkin C., De Trez M., Delemotte M., Johnson S.P. (2024). A systematic review of global marine mammal rehabilitation and refloating, 2000–2023. Sustainability.

[3] Hartman K.H., Yanong R.P., Harms C.A., Lewbart G.A. (2006). The future of training for aquatic animal health veterinarians. J. Vet. Med. Educ..

[4] Doneley B., Carmel B., Doneley B., Monks D., Johnson R., Carmel B. (2018). The Reptile Consultation. Reptile Medicine and Surgery in Clinical Practice.

[5] Harkewicz K.A. (2001). Dermatology of reptiles: A clinical approach to diagnosis and treatment. Vet. Clin. North Am. Exot. Anim. Pract..

[6] Govett P.D., Harms C.A., Linder K.E., Marsh J.C., Wyneken J. (2004). Effect of four different suture materials on the surgical wound healing of loggerhead sea turtles, *Caretta caretta*. J. Herpetol. Med. Surg..

[7] Disclafani R., Galluzzo P., Schirò G., Vazzana I., Lomonaco C., Monteverde V., Dara S. (2024). Evaluation of biochemical parameters in *Caretta caretta* sea turtles. Vet. Sci..

[8] Tauer A.M., Liles M.J., Chavarría S., Valle M., Amaya S., Quijada G., Seminoff J.A. (2017). Hematology, biochemistry, and toxicology of wild hawksbill turtles (Eretmochelys imbricata) nesting in mangrove estuaries in the eastern Pacific Ocean. bioRxiv.

[9] Noonburg G.E. (2005). Management of extremity trauma and related infections occurring in the aquatic environment. J. Am. Acad. Orthop. Surg..

[10] Higgins J.L., Hendrickson D.A. (2013). Surgical procedures in pinniped and cetacean species. J. Zoo Wildl. Med..

[11] Chittick E.J. (2003). Considerations in Sea Turtle Anesthesia and Surgery.

[12] Scarabelli S., Di Girolamo N. (2022). Chelonian sedation and anesthesia. Vet. Clin. Exot. Anim. Pract..

[13] Mitchell M.A., Diaz-Figueroa O. (2004). Wound management in reptiles. Vet. Clin. North Am. Exot. Anim. Pract..

[14] Fillman H., Riquelme P., Sullivan P.D., Mansoor A.M. (2020). Aseptic abscess syndrome. BMJ Case Rep..

[15] McCracken H., Carmel B., Chitty J., Doneley B., Johnson R., Lennox A.M., Monks D., Olsson A., Doneley B., Monks D., Johnson R., Carmel B. (2018). Differential Diagnoses: A Problem-Based Approach. Reptile Medicine and Surgery in Clinical Practice.

[16] Cushing A., Pinborough M., Stanford M. (2011). Review of bacterial and fungal culture and sensitivity results from reptilian samples submitted to a UK laboratory. Vet. Rec..

[17] Williams S.R., Sims M.A., Roth-Johnson L., Wickes B. (2012). Surgical removal of an abscess associated with *Fusarium solani* from a Kemp’s ridley sea turtle (*Lepidochelys kempii*). J. Zoo Wildl. Med..

